# Food Inequality Negatively Impacts Cardiac Health in Rabbits

**DOI:** 10.1371/journal.pone.0003705

**Published:** 2008-11-11

**Authors:** Fatemeh Heidary, Mohammad Reza Vaeze Mahdavi, Farshad Momeni, Bagher Minaii, Mehrdad Rogani, Nader Fallah, Roghayeh Heidary, Reza Gharebaghi

**Affiliations:** 1 Physiology Department, Shahed University, Medical School, Tehran, Iran; 2 Economy Department, Allameh Tabatabaei University, Tehran, Iran; 3 Histology Division, Anatomy Department, Medical School, Tehran University of Medical Sciences, Tehran, Iran; 4 Statistical Analysis Division, Social Medicine Department, Shahed University, Tehran, Iran; 5 Middle East Cancer Institute, Tehran, Iran; Georgia State University, United States of America

## Abstract

**Background:**

Individuals with lower socioeconomic status experience higher rates of mortality and are more likely to suffer from numerous diseases. While some studies indicate that humans who suffer from social inequality suffer generally worse health, to our knowledge no controlled experiments of this nature have been done in any species. Lipofuscin is a highly oxidized cross-linked aggregate consisting of oxidized protein and lipid clusters. This eminent terminal oxidation outcome accumulates within cells during aging process.

**Methodology/Principal Findings:**

Thirty two rabbits were assigned into four groups randomly of eight each. The first group encountered food deprivation for eight weeks and was kept in an isolated situation. The second group was food deprived for eight weeks but encountered to other groups continuously. The third group suffered two weeks of deprivation and then received free access to food. The fourth group had free access to diet without any deprivation. All hearts were removed for histopathological evaluation. Cross-sections of hearts were examined by light microscopy for the presence of yellow-brown Lpofuscin pigment granules. Here we show that relative food deprivation can cause accumulation of Lipofuscin pigmentation. We find that cardiac Lipofuscin deposition increases the most in the inequitable condition in which food deprived individuals observe well-fed individuals.

**Conclusions/Significance:**

Our findings demonstrate that a sense of inequality in food intake can promote aging more than food deprivation alone. These findings should be considered as a basis for further studies on the physiological mechanisms by which inequality negatively impacts health and well-being.

## Introduction

The relationship between socioeconomic status (SES) and health is well defined. Individuals with lower SES experience higher rates of mortality and are more likely to undertaking numerous health conditions. This so-called “social gradient” in health has been observed across different time periods and age groups using a extensive range of SES indicators, health measures, and methodologies [1], [2].

The purpose of this experiment was to study the effect of food intake inequality in accumulation of Lipofusion pigmentation in the rabbit heart. To our knowledge, this is the first study to examine a specific physiological outcome of social inequitably, in this case, the interaction of food intake inequality status and cardiovascular reactivity.

One of the highlights of postmitotic aging is the intracellular accumulation of highly oxidized and cross-linked proteins, known as lipofuscin [3], [4]. Proteins within Lipofuscin are linked by intramolecular and intermolecular cross-links. Many of these cross-links are caused by nonproteineous compounds including oxidation products [5], [6]. The intracellular formation of Lipofuscin is a complex arrangement of reactions involving numerous cellular compartments and enzymes. The intracellular rate of Lipofuscin formation is negatively correlated with the life expectancy of a postmitotic cell and enhances with age [7], [8]. It is understood that, the higher the rate of intracellular Lipofuscin accumulation over the time, the shorter the potential life time of the cell [9]. There is a considerable body of evidence indicating that oxidative stress is a causal factor both in Lipofuscinogenesis as well as in aging [10].

## Results

**Figure 1:**
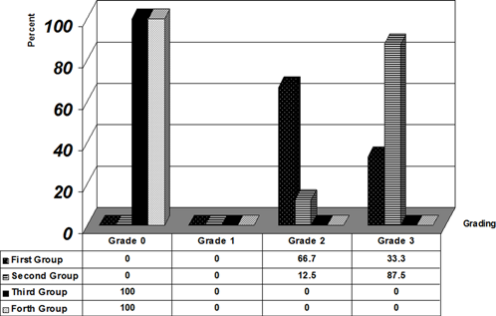
Percentage of Lipofuscin pigmentation between groups.

**Figure 2:**
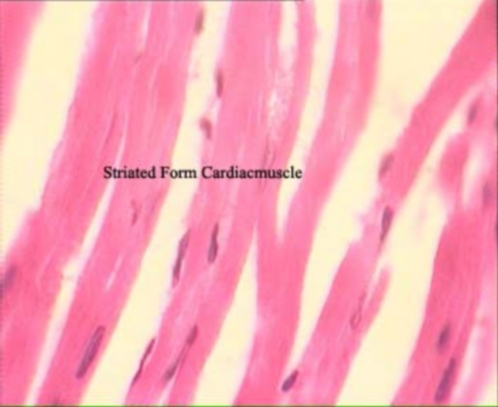
Longitudinal section through the heart of a rabbit showing normal structure of cardiac muscle in the control group.

**Figure 3:**
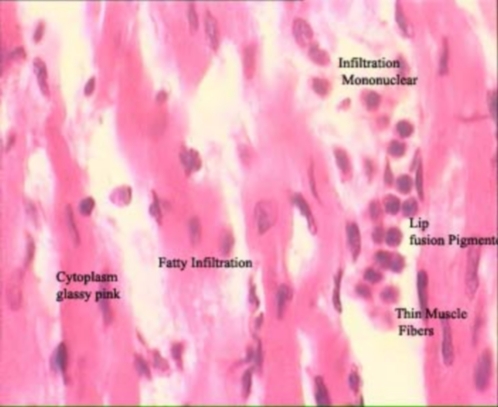
Longitudinal section through cardiac muscle showing Lipofuscin pigmentation, interstitial oedema, infiltration mononuclear, fibrosis and fatty infiltration in the group suffered from inequality.

Statistical analysis by Kruskal Wallis test showed significant differences between all groups (P<0.05). Further analysis using Mann Whitney test showed significant differences between the first and the second group (P<0.01; Table 1). This is a remarkable increase in the Lipofusion pigmentation accumulation and much more significant damage in the animals encountering the inequality situation (second group) rather than deprivation alone (first group; see Figure 1 shows percentage of damage between groups). In the control animals (fourth group), histopatholoical evaluation showed normal structure and none of the histopathologic changes were recorded, which was significantly different from the experimental (inequity) animals (Figure 2 and 3).

**Table 1:** Mean and standard deviation of Lipofuscin pigmentation between groups (P<0.05).

	First group	Second group	Third group	Fourth group
Mean	2.33	2.87	0.00	0.00
Standard Deviation	0.51	0.35	0.00	0.00

**First group:** Eight weeks suffered food deprivation in isolated situation.

**Second group:** Eight weeks suffered food deprivation but encountered to third and fourth groups (inequality situation).

**Third group:** Two weeks food deprivation then encountered to six weeks free access to food, not isolated.

**Fourth group:** Free access to food, not isolated.

## Discussion

Some empirical studies have indicated that animal models, like people, respond negatively to inequity [11]. Brosnan and de Waal showed that monkeys refused to participate if they witnessed a conspecific achieve a more attractive reward for equal endeavor, an effect amplified if the partner obtained such a reward without any effort at all. These feedbacks indicate the presence of an aversion to inequality in animals, and may support an early evolutionary origin of inequity aversion [12]. It has been established that Cynomolgus Macaques monkeys, fed an atherogenic chow and exposed to an emotionally stressful social situation, develop greater coronary heart disease rather than monkeys in a stable social condition. These studies suggest that behavioral factors can promote disease progression [13], [14], [15].

An extensive body of evidence from animal models reveals that chronic psychosocial stress can lead, probably via a mechanism involving excessive sympathetic nervous system establishment, to exacerbation of coronary artery atherosclerosis as well as to transient endothelial dysfunction. Supports from animal models also revealed that psychosocial stress reliably induces hypercortisolemia and excessive adrenergic activation in premenopausal females, leading to accelerated atherosclerosis [16].

Presumably, behavioral situation would potentiate disease through associated perturbations of the body's fundamental axes of neuroendocrine response, such as the hypothalamic–pituitary–adrenocortical and sympatheticadrenomedullary systems [14], [17]. Our study expands on this knowledge by demonstrating that such stress may be caused by comparisons of one's own outcomes to those of others. This is potentially critical for the health of social species, including humans, whose outcomes may differ between individuals.

It is well understood that Lipofuscin pigmentation is one of the most important characteristics of aging [9] and is responsible for ‘brown atrophy’ of the heart in gross pathology [18]. It might be worthwhile to mention that neuronal lipofuscin accumulation has been found to be increased in animals placed under restraint stress [19], increasing the plausibility that, in this study, a psychological stressor led to the observed changes.

There have been some studies indicating that humans who suffer from social inequality suffer generally worse health conditions mainly cardiovascular diseases [20], [21], [22], but nothing specific and no controlled experiments of this nature. To our knowledge these results are the first evidence that food inequality in animal models can cause any kind of diseases. Our results demonstrate that a sense of inequality in food intake, rather than food deprivation alone, could promote aging. Given the above facts, it is tempting to speculate that psychological changes may induce these variations, but more studies on this subject are needed.

In conclusion, these findings add to the body of evidence that food deprivation and inequality in food intake are highly important in the presence of Lipofusion pigmentation. Age related changes in food deprivation and food intake inequality may contribute to susceptibility to stressful situations for animals. These findings should serve as a basis for further studies to identify the causes of these variations and the major mechanisms underlying physiological responses to the stress of inequality.

## Materials and Methods

All procedures in this study were conducted in accordance with protocols approval by the Animal Care and Ethical Committee of Shahed University, Tehran, Iran. Thirty-two New Zealand rabbits (2.5 months old) obtained from Razi Institute, Hesarak, Karaj, Iran. All animals exposed to 12-hour light and dark conditions (lights on at 7 AM), housed in individual cages (6 Sq ft) and fed standard rabbit chow and tap water.

### Experimental design

All animals had free access to water during study. After acclimation procedures, rabbits were assigned into four groups randomly of eight each as below:

The first group encountered food deprivation (access to 33% of normal diet) for eight weeks. This group was kept in an isolated situation. The second group was food deprived for eight weeks but encountered to other groups continuously during study (the third and fourth groups). The third group suffered two weeks of deprivation and then received free access to food for another six weeks. The fourth group had free access to diet without any deprivation. At the end of the experiment, animals were sacrificed under ether anesthesia and their hearts were removed for histopathological evaluation.

### Histopathology

After euthanasia, heart were removed through a midline sections and washed once in phosphate-buffered saline to remove blood. Tissues were placed in a 10% solution of buffered formalin. Samples were coded, and all histomorphometric procedures were performed in a blind fashion. After gross examination, tissues were paraffin-embedded, sectioned at 3 micro meters, and stained with hematoxylin-eosin. To assess cardiac Lipofuscin pigmentation, cross-sections of hearts were examined by light microscopy for the presence of yellow-brown Lpofuscin pigment granules. All samples were graded double blindly from 0 to 3 histopathologically for expression of Lipofusion pigmentation by an expert histopathologist. The severity of microscopic lesions graded based on the following numerical scale: 0(absence), 1 (minimal), 2 (mild), 3 (severe).

### Statistical Methods

Kruskal Wallis and Mann Whitney tests were used for statistical evaluation through SPSS software version 11. Results of histopathological staining used to characterize cardiac Lipofuscin are listed in Table 1 as the mean and standard deviation between groups.
