# Correction: Food Inequality Negatively Impacts Cardiac Health in Rabbits

**DOI:** 10.1371/annotation/170470ba-f544-43dd-992e-52a06d3b41a4

**Published:** 2012-10-26

**Authors:** Fatemeh Heidary, Mohammad Reza Vaez Mahdavi, Farshad Momeni, Bagher Minaii, Mehrdad Rogani, Nader Fallah, Roghayeh Heidary, Reza Gharebaghi

There was a typographical error in the second author's name. The correct spelling is Mohammad Reza Vaez Mahdavi. The correct citation is:

Heidary F, Vaez Mahdavi MR, Momeni F, Minaii B, Rogani M, et al. (2008) Food Inequality Negatively Impacts Cardiac Health in Rabbits. PLoS ONE 3(11): e3705. doi:10.1371/journal.pone.0003705 

